# Cellular localization and regulation of receptors and enzymes of the endocannabinoid system in intestinal and systemic inflammation

**DOI:** 10.1007/s00418-018-1719-0

**Published:** 2018-09-08

**Authors:** Magdalena Grill, Carina Hasenoehrl, Melanie Kienzl, Julia Kargl, Rudolf Schicho

**Affiliations:** 1 Pharmacology Section, Otto Loewi Research Center, Medical University of Graz, Universitätsplatz 4/I, 8010 Graz, Austria; 2 BioTechMed Graz, Graz, Austria

**Keywords:** Cannabinoid receptors, GPR55, CB_1_, CB_2_, MGL, In situ hybridization

## Abstract

Surveys suggest that *Cannabi*s provides benefit for people with inflammatory bowel disease. However, mechanisms underlying beneficial effects are not clear. We performed in situ hybridization RNAscope^®^ combined with immunohistochemistry to show cell-specific distribution and regulation of *cannabinoid receptor 1* and *2* (*CB*_*1*_, *CB*_*2*_), *G protein-coupled receptor 55* (*GPR55*), and *monoacylglycerol lipase* (*MGL*) mRNA in immune cells using murine models of intestinal and systemic inflammation. In healthy animals, the presence in enteric ganglia is high for *CB*_*1*_ mRNA, but low for *CB*_*2*_ and *GPR55* mRNAs. *MGL* mRNA is predominant throughout the intestinal wall including myenteric neurons, epithelium, circular and longitudinal muscular layers, and the lamina propria. Within the immune system, B220^+^ cells exhibit high gene expression for *CB*_*2*_ while the expression of *CB*_*2*_ in F4/80^+^ and CD3^+^ cells is less prominent. In contrast, *GPR55* mRNA is highly present in F4/80^+^ and CD3^+^ cells. qRT-PCR of total colonic segments shows that the expression of *GPR55* and *MGL* genes drops during intestinal inflammation. Also at cellular levels, *GPR55* and *MGL* gene expression is reduced in F4/80^+^, but not CD3^+^ cells. As to systemic inflammation, reduced gene expression of *MGL* is observed in ileum by qRT-PCR, while at cellular levels, altered gene expression is also seen for *CB*_*1*_ and *GPR55* in CD3^+^ but not F4/80^+^ cells. In summary, our study reveals changes in gene expression of members of the endocannabinoid system in situ attesting particularly GPR55 and MGL a distinct cellular role in the regulation of the immune response to intestinal and systemic inflammation.

**Electronic supplementary material:**

The online version of this article (10.1007/s00418-018-1719-0) contains supplementary material, which is available to authorized users.

## Introduction

Knowledge of the endocannabinoid system (ECS) has immensely grown in the past few years. Its members, i.e., cannabinoid receptors, endocannabinoids and the enzymes that synthesize/degrade endocannabinoids, have become highly investigated targets [rev. in (Pertwee 2015)]. This molecular network has been recently coined “endocannabinoidome”, as more components have been added lately (Di Marzo and Piscitelli 2015). Apart from the brain, the gastrointestinal (GI) tract is one of the organs that contains key players of the ECS, including cannabinoid receptors (CB) 1 and 2 (CB_1_, CB_2_), G protein-coupled receptor 55 (GPR55) and the 2-arachidonoylglycerol (AG)-degrading enzyme monoacylglycerol lipase (MGL) [rev. in (Hasenoehrl et al. 2016)]. Functional tasks of the ECS in the GI tract comprise the control of the epithelial barrier (Cani et al. 2016; Karwad et al. 2017), the maintenance of immune homeostasis (Acharya et al. 2017), the regulation of motility, and the protection against inflammatory attacks (Schicho and Storr 2010). However, a clear understanding of how the ECS is regulated and which cell populations express these molecules during disease in situ is lacking. The GI tract is a heterogeneous organ, and therefore, accommodates the ECS in various cell types. CB_1_ and CB_2_ receptors, for instance, have been reported in the enteric nervous system and in epithelial and lamina propria cells (Wright et al. 2005, 2008). GPR55 has been located in enteric neurons and the colonic mucosa (Li et al. 2013). A recent study reported that GPR55 is involved in intraepithelial lymphocyte migration (Sumida et al. 2017). Little is known, however, about the cellular localization of GPR55 in the adaptive and innate immune system of the inflamed gut.

Much of the research progress on the ECS is hampered by a lack of specific tools, especially of antibodies against GPR55 and CB_2_, to detect their localization in different cell populations of the gut. As shown in several reports, antibodies against the CB_2_ receptor are likely unspecific (Baek et al. 2013; Cecyre et al. 2014; Marchalant et al. 2014). Commercially available antibodies against CB_1_ have also been evaluated, showing that unspecific staining may be an issue with CB_1_ antibodies as well (Grimsey et al. 2008). In intestinal tissue, commercially available GPR55 antibodies have been used for immunohistochemistry with more or less strict controls (Galiazzo et al. 2018; Li et al. 2013; Lin et al. 2011). However, most of them show a band in GPR55^−/−^ mice at the right molecular weight (see online resource Fig. 1). The strongest indication for antibody specificity, therefore, is the absence of staining in the respective knockout mouse.

In this context, in situ hybridization (ISH) has proved to be a most valuable and highly specific alternative to demonstrate the presence and regulation of CB receptors (Li and Kim 2015). As PCR analysis of whole intestinal tissue does not reveal in which cells CB receptor regulation occurs, ISH can be combined with immunohistochemistry (IHC) in co-localization studies to identify the cells in which regulation of receptors takes place. We, therefore, localized and evaluated *CB*_*1*_, *CB*_*2*_, *GPR55* and *MGL* gene expression (the official symbols of encoding genes are termed *Cnr1, Cnr2, Gpr55 and Mgll*) in mouse models of intestinal and systemic inflammation by a novel ISH technique (RNAscope^®^) with emphasis on the localization of *GPR55*. For co-localization with distinct immune cell populations, we used established T-cell, B-cell and macrophage markers. Intestinal inflammation in mice was induced by adding 2.5% dextran sulfate sodium (DSS) to the drinking water. DSS disrupts the epithelial barrier in the colon causing inflammation reminiscent of human ulcerative colitis (Eichele and Kharbanda 2017). Systemic inflammation was performed by treatment with lipopolysaccharide (LPS), a model that leads to intestinal barrier dysfunction (Han et al. 2004; Kimura et al. 1997), epithelial injury and a change in T cell populations (Liu et al. 2009). The specificity of the probes was tested in sections of the respective knockout mice (CB_1_^−/−^, CB_2_^−/−^, GPR55^−/−^, and MGL^−/−^).

## Materials and methods

### Animals

C57BL/6 mice obtained from Charles River (Sulzfeld, Germany) were used for the experiments. CB_1_^−/−^ and MGL^−/−^ mice were kindly provided by A. Zimmer and R. Zimmermann, respectively (Hasenoehrl et al. 2018; Taschler et al. 2011). CB_2_^−/−^ mice were purchased from Jackson Laboratory (Bar Harbor, ME, USA) and GPR55^−/−^ animals from the Mutant Mouse Resource & Research Center (MMRRC; University of North Carolina, Chapel Hill, NC; USA) (Hasenoehrl et al. 2018). Knockout mice with a C57BL/6 background served as negative controls for ISH and IHC. All mice were bred in our own facilities and housed in plastic cages with sawdust floors at constant temperature (22 °C) under a 12 h light/12 h dark cycle with standard lab food and water *ad libitum*. The experimental procedures were approved by the Austrian Federal Ministry of Science, Research and Economy (protocol numbers: BMWF- 66.010/0131-WF/V/3b/2014; BMWF-66.010/0044-WF/V/3b/2016) and performed in strict accordance with international guidelines.

### Treatment of animals

#### DSS-induced intestinal inflammation

Colitis was induced in C57BL/6 mice by adding 2.5% DSS (MP Biomedicals, Illkirch, France) to their drinking water for 5 days and animals were killed on day 8 (Stancic et al. 2015).

#### LPS-induced systemic inflammation

C57BL/6 mice were treated with an intraperitoneal injection of 0.83 mg/kg LPS (Farzi et al. 2015) (Sigma, Vienna, Austria) or with PBS alone (vehicle) and killed 4 h later.

### Tissue processing

Colon (DSS treatment) or ileum (LPS treatment) was harvested and cut in half. One piece of tissue was snap frozen in liquid N_2_ and stored at −80 °C until it was further processed for quantitative reverse transcription-polymerase chain reaction (qRT-PCR), the other half was fixed in acid free phosphate-buffered 10% formaldehyde solution (Roti^®^-Histofix 10%; pH 7, stabilized with methanol) for 16–24 h at room temperature.

### RNA isolation and qRT-PCR

RNA was extracted from tissue samples using TRIzol (Life Technologies). Samples from DSS-treated animals were purified in an additional step with the “RNA clean up” protocol from the RNeasy kit and subsequently subjected to the Oligotex mRNA Mini Kit (both Qiagen) to remove remaining DSS that could inhibit subsequent qRT-PCR steps. All samples were treated with the DNA Removal Kit DNA-free (Life Technologies) and 2 µg of total RNA were reverse transcribed to cDNA using High-Capacity cDNA Reverse Transcription Kit (Applied Biosystems, Carlsbad, USA).

Gene expression of *CB*_*1*_, *CB*_*2*_, *GPR55* and *MGL* was quantified using SsoAdvanced^™^ Universal SYBR^®^ Green Supermix (Bio-Rad, Vienna, Austria). Validated primers were purchased from Bio-Rad and relative gene expression was assessed according to the 2^−ΔΔ*C*q^ method.

### In situ hybridization (ISH) and immunohistochemistry (IHC)

Freshly harvested and fixed tissue was further processed for paraffin embedding according to standard procedures. Tissue was cut in 5 µm sections, baked at 60 °C for 1 h, de-waxed and rehydrated.

### ISH

RNAscope^®^ is an advanced ISH method where two adjacent probes (the so-called ZZ probes) are needed to bind to the target sequence to develop a signal. This method provides the possibility of detecting a low number of mRNAs in peripheral tissue due to a decreased background noise (Wang et al. 2012).

20 ZZ probes for murine *CB*_*1*_ (targeting bases 701–1792 of NM_007726.3), *CB*_*2*_ (targeting bases 291–719 of NM_009924.3), *GPR55* (targeting bases 2–907 of NM_001033290.2) and 3 ZZ probes for *MGL* (targeting bases 703–849 of NM_001166251.1) (all purchased from Advanced Cell Diagnostics, ACD, Newark, USA) were used to detect the corresponding mRNAs in murine intestinal or systemic inflammation models. ISH (RNAscope^®^ 2.5 HD brown or red kit for *CB*_*1*_, *CB*_*2*_ and *GPR55* and BASEscope^™^ red kit for *MGL*, ACD) was performed according to manufacturer’s protocol. In brief, tissue sections were treated with H_2_O_2_ for 10 min, target retrieval was performed using the Brown FS3000 food steamer for 15 min, each step followed by washes in H_2_O dest. The next day, sections were digested with Protease Plus (for *CB*_*1*_, *CB*_*2*_ and *GPR55*) or Protease IV (*MGL*) at 40 °C for 20 min, washed, followed by incubation with the corresponding probes at 40 °C for 2 h. The procedure was continued according to the manufacturer’s protocol for RNAscope^®^ or BASEscope™ (ACD). *CB*_*1*_, *CB*_*2*_, *GPR55* and *MGL* were stained using 3,3′-diaminobenzidine (DAB; for brightfield) or FastRed (for brightfield and fluorescence, both dyes provided by ACD). Colonic or ileal sections from treated and untreated C57BL/6 mice and the corresponding knockout controls were put on one slide for comparison.

### IHC

Antibodies against cell markers including CD3, CD4, CD8 and FoxP3 (for T-cell subtypes), F4/80 (for monocytes/macrophages), CD45R-B220 (for B lymphocytes), and neurofilament H and synaptophysin (for neuronal structures [axons/synapses]) were used to determine cell types co-localizing with mRNAs (see Table 1).

**Table 1:** Antibodies used in this study

Antigen/antibody (clone/cat no)	Provider	Specificity shown by	Dilution	Host/isotype
CD3 (ab5690)	Abcam, Cambridge, UK	WB (data sheet)	1:1000	Rabbit IgG pAb
CD4 (EPR19514/ ab183685)	Abcam, Cambridge, UK	WB, IP (data sheet)	1:1000	Rabbit IgG mAb
CD8 (ab203035)	Abcam, Cambridge, UK	WB (data sheet)	1:2000	Rabbit IgG pAb
FoxP3 (D6O8R/ #12653)	Cell Signaling Technology, Danvers, MA, USA	Flow cytometry (data sheet)	1:200	Rabbit IgG mAb
F4/80 (BM8/ sc-52664)	Santa Cruz Biotechnologies, Dallas, TX, USA	Comparison to CD68 (Shi 2010), data sheet reference)	1:200	Rat IgG2a, mAb
CD45R-B220 (RA3-6B2/ 14-0452-82)	Thermo Fisher Scientific, Waltham, MA, USA	Flow cytometry, WB (data sheet)	1:250	Rat IgG 2a, kappa, mAb
Neurofilament H(ab8135)	Abcam, Cambridge, UK	WB (data sheet)	1:2000	Rabbit IgG, pAb
Synaptophysin (SP11/ MA5-14532)	Thermo Fisher Scientific, Waltham, MA, USA	WB (data sheet)	1:250	Rabbit IgG, mAb
Biotinylated anti-rabbit IgG	Vector laboratories, Burlingame, CA, USA	Omission of primary Ab	1:200	Goat IgG
Biotinylated anti-rat IgG (BA-9401)	Vector laboratories, Burlingame, CA, USA	Enzyme immunoassay (data sheet)	1:200	Goat IgG
Anti-rabbit Alexa Fluor® 488	Thermo Fisher Scientific, Waltham, MA, USA	Omission of primary Ab	1:500	Goat IgG

*WB* Western blot, *IP* immunoprecipitation, *m* monoclonal, *p* polyclonal, *Ab* antibody

Tissue sections were blocked in 0.1 M PBS containing 0.3% Triton X-100 and 5% goat serum (Sigma-Aldrich/ Merck, Darmstadt, Germany). First antibody in 0.1 M PBS containing 0.3% Triton X-100 and 1% goat serum was applied over night at 4 °C, IHC was performed using the Vectastain^®^ABC kit and Vector^®^ VIP HRP substrate kit (both Vector Laboratories) according to the manufacturer’s protocol. Sections were counterstained with 1:5 dilutions of Gill’s II Hematoxylin or with Methyl green, washed, dried and mounted with Vectamount mounting medium (Vector Laboratories).

### Microscopy

Brightfield images were taken using a Zeiss Axiophot (100x/1.30 Plan-Neofluar objective with oil immersion; Carl Zeiss AG, Oberkochen, Germany) equipped with a high resolution CCD camera (*coolsnap cf* from Photometrics^®^, Tuscon, AZ, USA; images: 1392 × 1040 pixels; 24 bit) and MCID™ Analysis software 7.0 (InterFocus Imaging Ltd, Linton, England), or an Olympus BX41 microscope (objectives: 20x/0.75, UPlanSApo; 40x/0.95, UPlanSApo; 100x/1.40, UplanSApo with oil immersion) and an Olympus UC 90 digital camera; images: 1688 × 1353 pixel; 24 bit connected with Olympus CellSense^®^ standard 1.17 imaging software (Olympus, Vienna, Austria).

Fluorescence images were taken by an Olympus IX70 (objectives: 10x UPlanFl) connected with an Olympus MT20 light source (150W xenon arc burner) and a Hamamatsu ORCA-ER digital camera (1344 × 1024 pixels; Hamamatsu Photonics K.K., Japan). Olympus xcellence^®^ imaging analysis software 1.1 was used for acquiring images (1344 × 1024 pixels, 24 bit). The following fluorescence filters were used: for Fast Red (U-M41007 cube): DM568, excitation filter 540–560, barrier filter 575–645; for Alexa 488 (modified U-MNIBA cube): DM 505, excitation filter BP470-490, barrier filter BA515-550; for DAPI (Olympus DAPI Filter MT20): DM 409, excitation filter 378/52 (MT20 light source), barrier filter HC Quadband Filter 432. Contrast, brightness and color balance of images were adjusted using Corel Photo Paint^®^.

### Quantification of ISH gene expression

Tissues to be compared were mounted on one slide to be processed together for ISH and IHC.

A semi-quantitative histological scoring method was chosen based on ACD scoring criteria for RNAscope^®^ to count and compare gene expression in different cell types and between control and diseased animal tissue. In brief, about 100 cells per tissue/mouse (3 mice/ treatment) were evaluated. Only cells with a visible nucleus and a clearly confined cell body were taken into account. Dots in each cell were counted and cells were allocated to one of five groups. Group 0 means no expression, group 1 is 1–3 dots, group 2 is 4–9 dots or 1 cluster, group 3 is 10–15 dots or few clusters, group 4 is > 15 dots or many clusters. The columns in the graphs indicate the percentage of cells in each group (sum of all groups per treatment equals 100%).

### Statistics

ISH: Data are shown as mean +/– standard error of the mean (SEM) with three mice (ISH) or five–ten mice (qRT-PCR) per experimental group. Statistical analysis was done using GraphPad Prism 5.03 (GraphPad Software, La Jolla, CA, USA). Cell counts were compared by two-way analysis of variance followed by Bonferroni multiple comparison post hoc analysis. Gene expression was compared by unpaired two-tailed Student’s *t* test. *P* values < 0.05 were considered significant and denoted with 1, 2 or 3 asterisks when lower than 0.05, 0.01 or 0.001, respectively.

## Results

### Specificity of the ISH probes

ISH RNAscope^®^ and ISH/IHC were performed to determine detailed cellular localizations of mRNA and to investigate, if changes in gene expression by DSS and LPS treatment can be detected on a cellular level in situ. To evaluate the kit performance and the quality of the fixed tissue, we first performed ISH RNAscope^®^ using the positive (PPIB) and negative (bacterial dapB) probes provided by the manufacturer. The positive probe produced visible ISH staining on all tissues, while the negative probe showed no ISH staining (not shown). Pretreatment conditions were adjusted to minimize tissue digestion and background while maintaining highest possible positive ISH signal. To confirm the specificity of the target probes, untreated CB_1_^−/−^, CB_2_^−/−^, GPR55^−/−^ and MGL^−/−^ mice were used and compared to healthy or treated wild type mice. CB_1_, CB_2_ and GPR55 probes, used with the standard RNAscope^®^ kit, showed no staining at all or only minimal background staining (see online resource Fig. 2). Initially, all RNAscope^®^ probes were detected using the brown detection kit. To better distinguish between ISH and IHC stainings, we switched to the red detection kit for some selected targets. *MGL*, detected by the highly sensitive red BASEscope™ kit, resulted in only minimal background staining in the corresponding knockout tissue.

#### Colon/ DSS-induced colitis

##### Localization and extent of *CB*_*1*_ gene expression markedly differs from *CB*_*2*_ gene expression in the murine colon

**Fig. 1:**
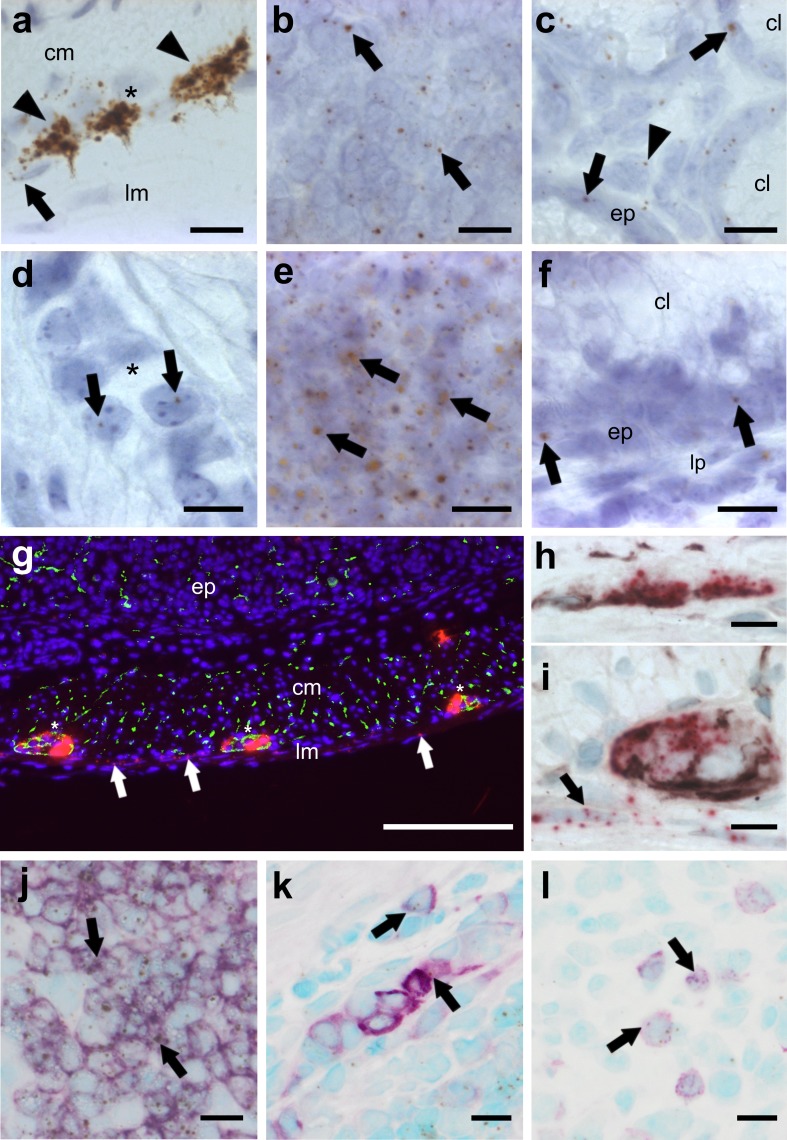
ISH staining of *CB*_*1*_ (**a**–**c, g**–**i**) and *CB*_*2*_ (**d**–**f, j**–**l**) mRNA in the colon of healthy mice. *CB*_*1*_ mRNA is strongly detected in neurons of the myenteric (**a** brown, DAB, arrowheads; **i** FastRed) and the submucosal plexus (**h** FastRed). *CB*_*1*_ gene expression is also found in the longitudinal muscle layer (**a, g, i***arrows*), in lymph follicles (**b** arrows) and to a lesser degree in epithelial cells (**c** arrows) as well as some cells of the lamina propria (**c**, arrowhead). In contrast, *CB*_*2*_ gene expression is scarcely found in the myenteric plexus (**d** arrows). Highest expression of the *CB*_*2*_ gene occurs in lymph follicles (**e** arrows), whereas low expression is seen in epithelial cells (**f***ep*) and the lamina propria (**f***lp*). *CB*_*1*_ mRNA fluorescence staining (FastRed) shows modest co-localization (yellow) with synaptophysin (green) in the myenteric plexus (**g** plexus denoted by asterisks). *CB*_*1*_ mRNA (FastRed) shows clear co-localization with neurofilament H (brown, VIP) in the submucosal (**h**) as well as in the myenteric (**i**) plexus. *CB*_*1*_ mRNA is also found in the longitudinal muscular layer where it does not co-localize with either of the neuronal markers (arrows in **g** and **i**). *CB*_*2*_ mRNA (brown, DAB) is predominantly detected in B220^+^ cells (purple, VIP) of lymph follicles (**j**) and only marginally in F4/80^+^ (**k**) or CD3^+^ (**l**) cells. Calibration bars: **a–f, h–l** 10 µm, **g** 200 µm; *cm* circular muscle layer, *ep* epithelium, *lp* lamina propria, *lm* longitudinal muscle layer, *cl* crypt lumen; *, myenteric ganglion; arrows point at representative cells expressing *CB*_*1*_ or *CB*_*2*_ genes

**Fig. 2:**
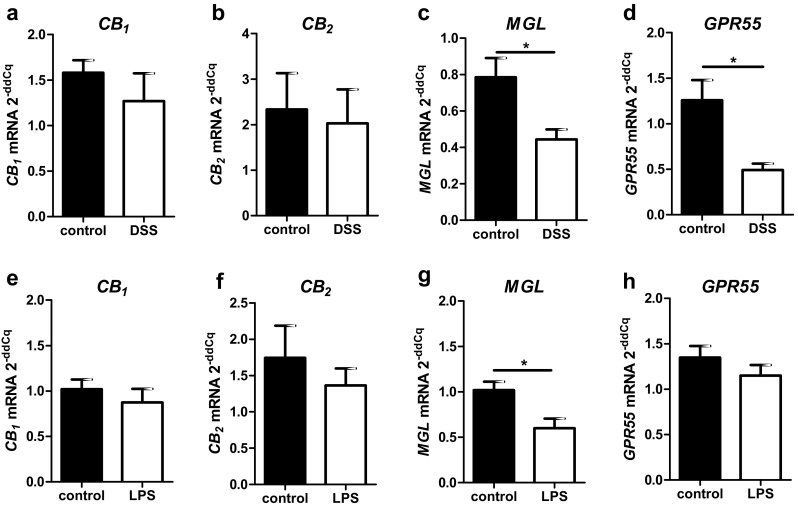
qRT-PCR evaluation of total mRNA levels of colon (**a**–**d**) and ileum (**e**–**h**) from healthy and diseased mice. While *CB*_*1*_ (**a**) and *CB*_*2*_ (**c**) gene expression are not significantly altered in mice after DSS treatment, *GPR55* (**b**) and *MGL* (**d**) gene expression are significantly reduced in DSS-induced colitis. In LPS-induced systemic inflammation, *CB*_*1*_, *CB*_*2*_ and *GPR55* mRNA levels do not differ from healthy tissue (**e–g**). In contrast, *MGL* mRNA levels are reduced in comparison to healthy animals (**h**)

The regulation of CB_1_ and CB_2_ at the cellular level in situ during inflammation is still unexplored and the localization of CB_2_ receptors in the colon has been conflicting so far. Therefore, *CB*_*1*_ and *CB*_*2*_ gene expression and localization in healthy and DSS-treated mice was investigated using ISH RNAscope^®^. In healthy tissue, *CB*_*1*_ gene expression was prominent in enteric ganglia (Fig. 1a), but was also seen in lymph follicles (Fig. 1b), and in epithelial and lamina propria cells (Fig. 1c). Contrary to *CB*_*1*_, *CB*_*2*_ mRNA was heavily expressed in lymph follicles (Fig. 1e) but only marginally in the myenteric plexus (Fig. 1d) and the epithelium (Fig. 1f) of healthy colon. qRT-PCR showed that *CB*_*1*_ and *CB*_*2*_ mRNA levels were not altered in total tissue of DSS-treated mice (Fig. 2a, b), as compared to healthy animals. Likewise, no differences in *CB*_*1*_ and *CB*_*2*_ mRNA levels were found on the cellular level using scoring criteria as described in the *Methods* (data not shown). To investigate if *CB*_*1*_ mRNA staining in the muscle layers was of neuronal or muscular origin, we stained for neuronal structures using synaptophysin and neurofilament H (NFH). Some *CB*_*1*_ mRNA co-localized with synaptophysin (Fig. 1g) and NFH in ganglia (Fig. 1h, i). *CB*_*1*_ mRNA was also found in the longitudinal muscle layer and did not co-localize with the neuronal markers (Fig. 1g, arrows), indicating *CB*_*1*_ gene expression in the longitudinal muscle cells themselves. *CB*_*2*_ mRNA highly co-localized with the B-cell marker B220^+^ (Fig. 1j). Some *CB*_*2*_ gene expression was also found in F4/80^+^ (Fig. 1k) and CD3^+^ cells (Fig. 1l).

##### The *MGL* gene is highly expressed in healthy and to a lesser degree in DSS-treated mice

**Fig. 3:**
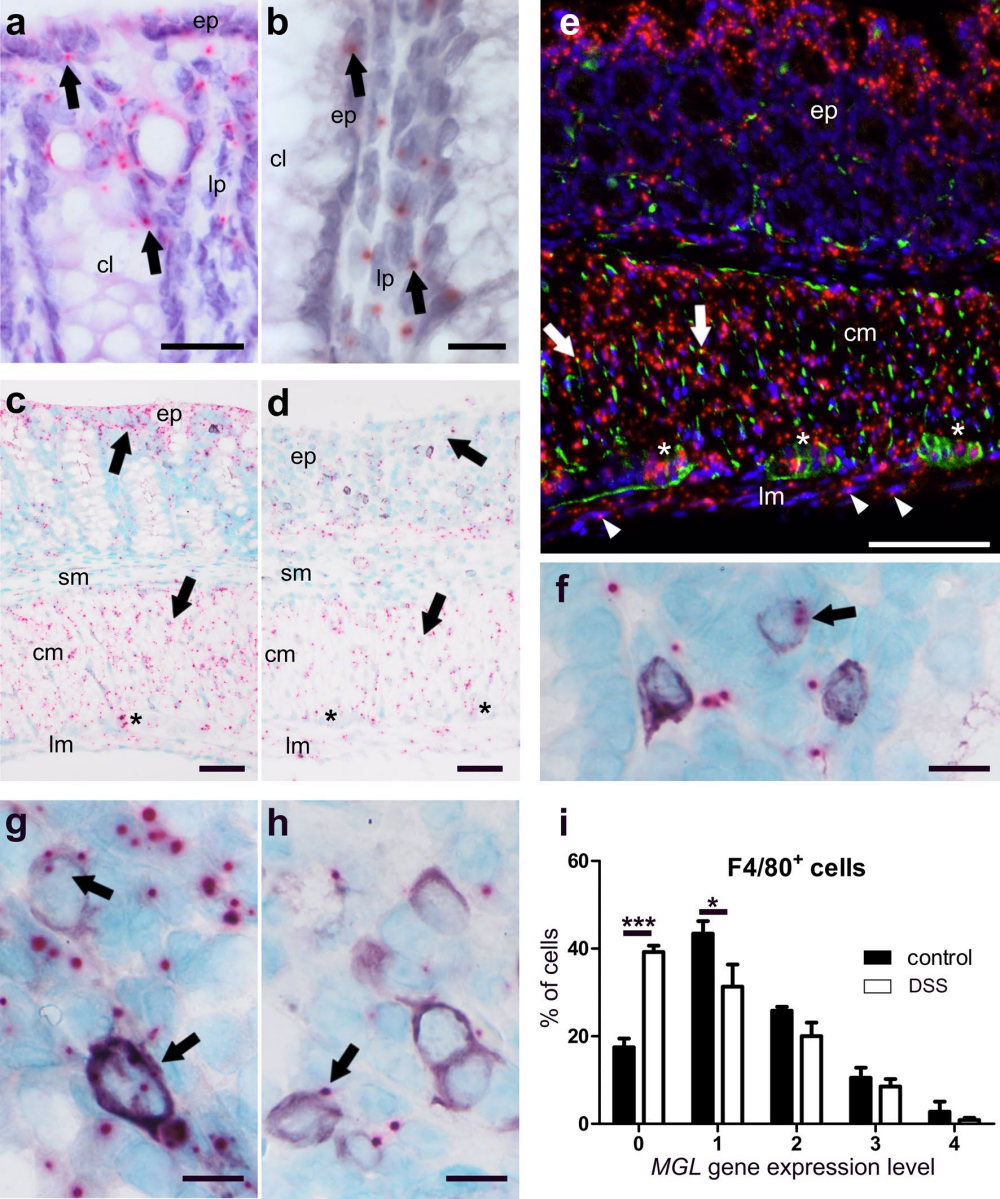
ISH staining of *MGL* mRNA in colon of healthy (**a**–**c, e**–**g**) and DSS-treated mice (**d, h**). *MGL* mRNA (FastRed) is seen in cells of the epithelium (*ep*) and the lamina propria (*lp*) (**a, b**), myenteric ganglia (**c***asterisk*), and in the circular (*cm*) and longitudinal (*lm*) muscle layer (**c**) of healthy colonic mucosa. In contrast, *MGL* gene expression drops throughout the whole colon after DSS treatment, particularly in the epithelium (*ep*) and the circular muscle layer (*cm*) (**d**, *arrows*). **c** and **d** Taken from ISH/IHC double staining of *MGL* mRNA (FastRed) and CD3 protein (purple, VIP). Immunofluorescence shows that *MGL* mRNA (red, FastRed) is abundantly stained in the circular (*cm*) as well as the longitudinal (*lm; arrowheads*) muscle layer (**e**). Co-localization of *MGL* mRNA with synaptophysin (green, Alexa 488) is rather low (yellow dots; *arrows*; **e**). There is only little *MGL* gene expression (pink, FastRed) in CD3^+^ cells (purple, VIP) (**f**). Gene expression of *MGL* in F4/80^+^ cells (purple, VIP) is decreased after DSS treatment (**h**) as compared to healthy animals (**g**). Quantification of *MGL* mRNA in F4/80^+^ cells in healthy versus diseased animals is shown in **i;** note the increased cell number in expression level 0 (no expression) and reduced number of cells in expression level 1. Calibration bar in **a** 20 µm; calibration bars in **b, f, g, h** 10 µm; calibration bars in **c, d** 50 µm; calibration bar in **e** 100 µm; *cm* circular muscle layer, *ep* epithelium, *lp* lamina propria, *lm* longitudinal muscle layer, *cl* crypt lumen, *sm* submucosa; *, myenteric ganglia. Arrows point at representative cells expressing *MGL* mRNA

*MGL* gene expression was found in colonic mucosa, epithelial cells, lamina propria cells, myenteric ganglia and particularly in muscle layers of healthy mice (Fig. 3a–c). In DSS-treated animals, qRT-PCR showed that overall *MGL* gene expression was reduced (Fig. 2c). This reduction was particularly visible in the epithelium and the circular muscle layer of ISH-stained colon sections (Fig. 3d). To investigate the source of *MGL* mRNA in the muscle layers, we performed double ISH/immunofluorescence with *MGL* and synaptophysin (Fig. 3e) or NFH (not shown). While little co-localization of *MGL* mRNA with synaptophysin was detectable (Fig. 3e, arrows), most of the *MGL* mRNA staining did not overlap with synaptophysin/NFH. To determine the cell type responsible for *MGL* gene expression in the lamina propria, we co-stained for *MGL*/CD3 (Fig. 3f) and F4/80 (Fig. 3g, h). While the majority of CD3^+^ cells were negative for *MGL* gene expression, over 80% of F4/80^+^ cells did express *MGL* in healthy animals (Fig. 3i).

##### *GPR55* mRNA is present in T-cells and macrophages of healthy murine colon

**Fig. 4:**
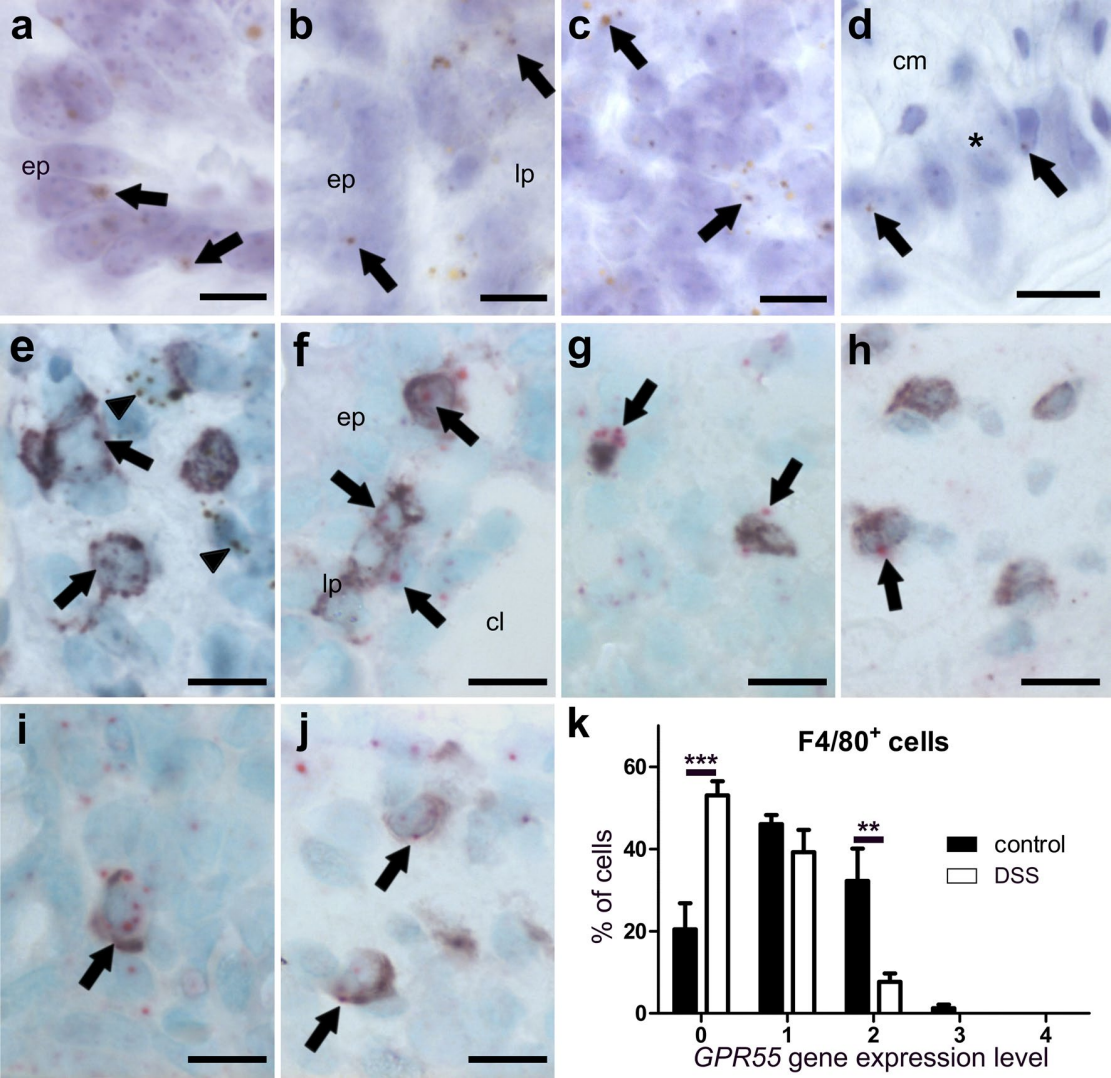
ISH staining of *GPR55* mRNA in colon of healthy (**a**–**i**) and DSS-treated mice (**j**). In the healthy colon, there is moderate expression of the *GPR55* gene in epithelial cells (**a**, *ep*), lamina propria cells (**b**, *lp*), lymph follicle (**c**), and very low expression in myenteric neuronal cells (**d** brown, DAB; representative *GPR55* mRNA staining is denoted by *arrows*). Co-localization of *GPR55* mRNA (brown, DAB) with CD3 (purple, VIP, *arrows*) in the lamina propria indicates *GPR55* gene expression in T-cells although staining is also seen outside CD3^+^ cells (**e***arrowheads*). *GPR55* mRNA (FastRed) can be found in all T-cell subpopulations investigated such as in CD4^+^ (**f**), FoxP3^+^ T regulatory (**g**), and CD8^+^ cells (**h**). In F4/80^+^ cells, *GPR55* mRNA levels are decreased after treatment with DSS (**j**) as compared to healthy mice (**i**); the corresponding quantification of cell counts is shown in (**k**). Calibration bars: 10 µm; *cm* circular muscle layer, *ep* epithelium, *lp* lamina propria, *lm* longitudinal muscle layer, *cl* crypt lumen; *, myenteric ganglion

*GPR55* gene expression was detected in epithelial cells (Fig. 4a), cells of the lamina propria (Fig. 4b) and lymph follicles (Fig. 4c). *GPR55* mRNA was only marginally found in neurons of the myenteric plexus (Fig. 4d). No *GPR55* gene expression was found in the muscular layers of the colon (not shown). To further determine the cell type of highly expressing cells in the lamina propria, we co-stained for T-cell markers and for the monocyte/macrophage marker F4/80 in healthy tissue. The *GPR55* gene was highly expressed in CD3^+^ (Fig. 4e) and F4/80^+^ cells (Fig. 4i, j). All subpopulations of T-cells were positive for *GPR55* mRNA staining, i.e., CD4^+^ (Fig. 4f), FoxP3^+^ T regulatory (Fig. 4g), and CD8^+^ cells (Fig. 4h).

##### *MGL* and *GPR55* gene expression is particularly altered in F4/80^+^ cells of DSS-treated mice

According to qRT-PCR, levels of *CB*_*1*_ and *CB*_*2*_ mRNAs from the entire colon wall did not significantly change in intestinal inflammation, whereas *GPR55* and *MGL* gene expression decreased in DSS-treated versus vehicle-treated mice (Fig. 2c, d). While part of this could be explained due to damaged epithelial cells, we asked whether reduction could have also occurred in immune cells. Interestingly, scoring of *GPR55* and *MGL* gene expression in CD3^+^ cells revealed no changes (not shown). However, *MGL* (Fig. 3h, i) and *GPR55* (Fig. 4j, k) clearly showed reduced gene expression in F4/80^+^ cells after DSS treatment as compared to healthy animals (*MGL*: Fig. 3g; *GPR55*: Fig. 4i).

#### Ileum/LPS-induced systemic inflammation

##### *CB*_*1*_ gene expression is slightly altered at the cellular level, whereas no changes for *CB*_*2*_ gene expression were observed in ileum after LPS treatment

**Fig. 5:**
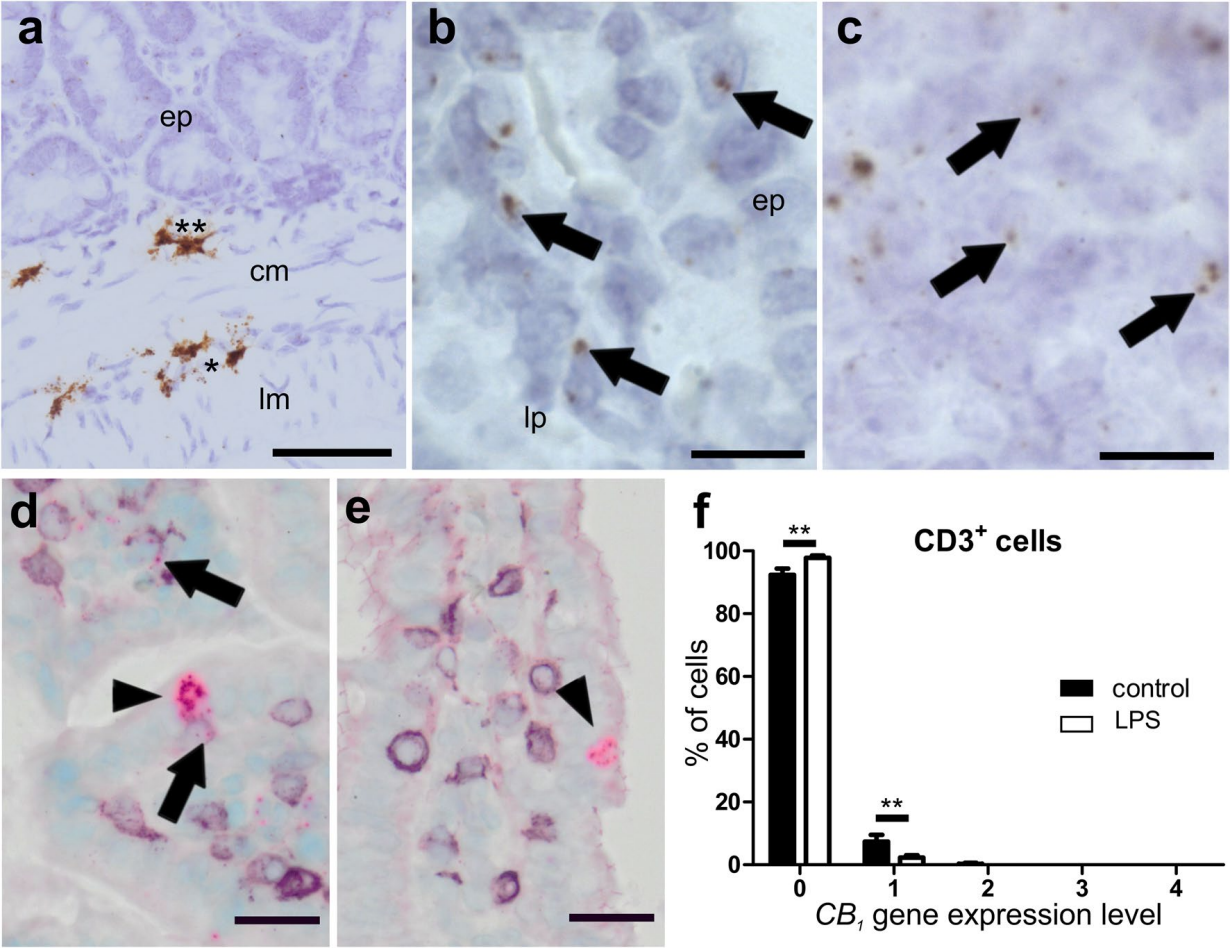
ISH staining of *CB*_*1*_ mRNA in ileum of healthy (**a**–**d**) and LPS-treated (**e**) mice. Strong *CB*_*1*_ mRNA ISH staining (brown, DAB) is seen in myenteric (*asterisk*) and submucosal ganglia (double *asterisk*) in the ileal wall of healthy mice (**a**). There is only low expression of *CB*_*1*_ mRNA (*arrows*) in the epithelium (**b***ep*) and the lamina propria (**b***lp*). High magnification of lymph follicles shows *CB*_*1*_ gene expression in healthy animals (**c**). High levels of *CB*_*1*_ mRNA (FastRed) are detected in isolated cells of the epithelium, probably neuroendocrine cells (*arrowheads*), from healthy (**d**) as well as LPS-treated (**e**) mice. Only marginal staining of *CB*_*1*_ mRNA is found in CD3^+^ cells (**d**, purple, VIP; *arrows*) that is even less in the ileum of LPS-treated animals (**e**). Quantification of *CB*_*1*_/CD3^+^ double staining is shown in **f**. *C*alibration bar in **a**: 50 µm; calibration bar in **b, c**: 10 µm; calibration bar in **d, e**: 20 µm; *cm* circular muscle layer, *ep* epithelium, *lp* lamina propria, *lm* longitudinal muscle layer; *, myenteric ganglion; **, submucosal ganglion

**Fig. 6:**
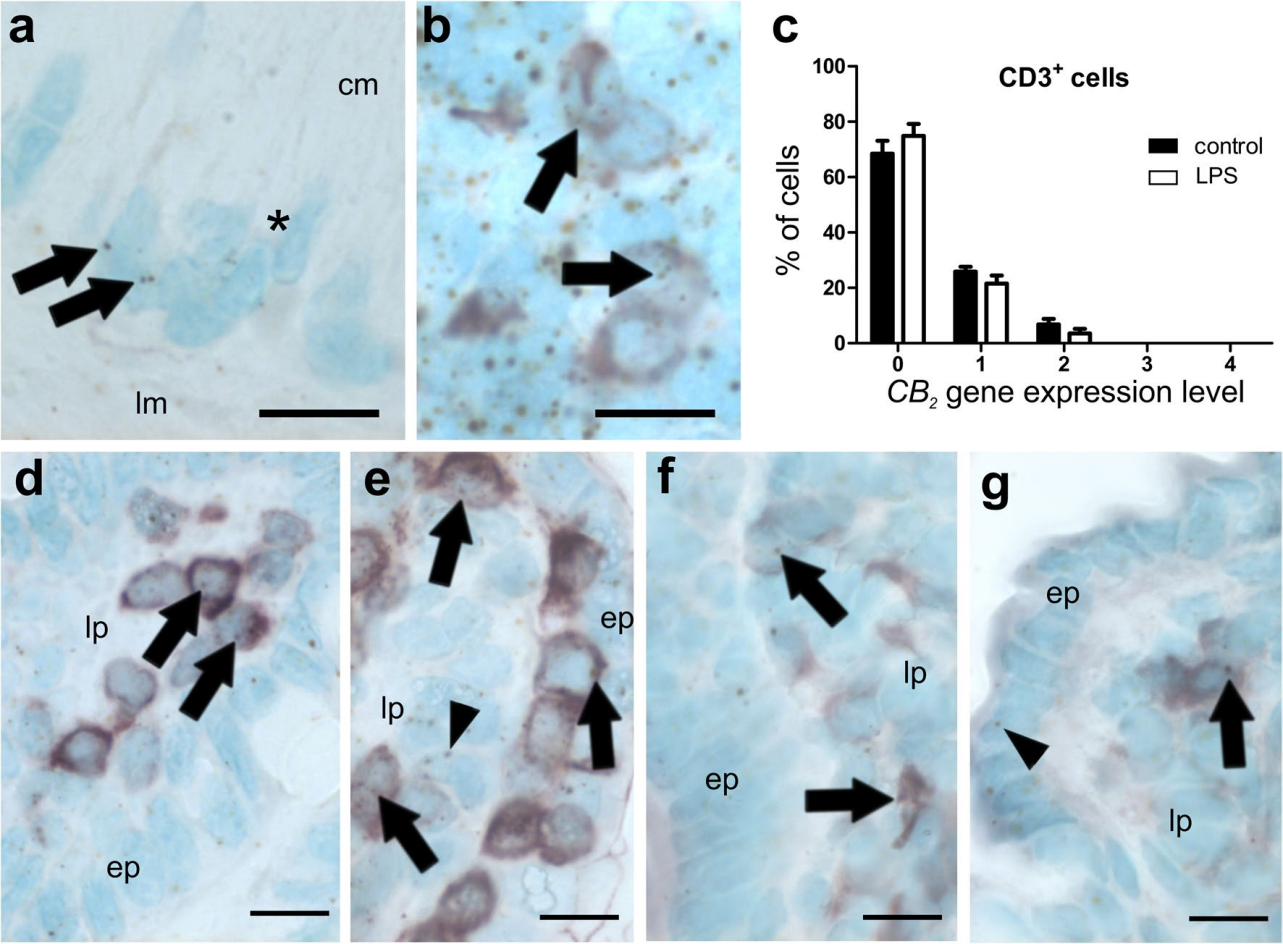
ISH staining of *CB*_*2*_ mRNA in ileum of healthy (**a, b, d, f**) and LPS-treated (**e, g**) mice. Healthy mice show very low gene expression of *CB*_*2*_ (brown, DAB) in myenteric ganglia of the ileum (**a** marked by *asterisk; arrows* point at mRNA staining). Compared to myenteric ganglia, cells in lymph follicles show widespread *CB*_*2*_ gene expression, some located in CD3^+^ cells (**b** arrows). The *CB*_*2*_ gene is also expressed in lamina propria CD3^+^ cells of healthy animals, although at a low level (**d** arrows point at expression in representative cells). Cells often line up underneath the epithelium (*ep*; **e**). Also CD3 negative cells expressing the *CB*_*2*_ gene are visible In the lamina propria (*lp*) (**e** arrowhead). In addition, cells stained for F4/80 show *CB*_*2*_ mRNA in healthy (**f**) and LPS-treated (**g**) mice. There is only marginal expression of *CB*_*2*_ in epithelial cells even after LPS treatment (**g**; *arrowhead*). Quantification of *CB*_*2*_ mRNA levels in CD3^+^ cells is shown in **c**, indicating that *CB*_*2*_ gene expression is not significantly altered after LPS treatment. Calibration bars: 10 µm; *ep* epithelium, *lp* lamina propria, *, myenteric ganglion

High *CB*_*1*_ gene expression was detected in the submucosal and the myenteric plexus (Fig. 5a) and in isolated cells of the epithelium, probably neuroendocrine cells (Fig. 5d). Low expression was found in the lamina propria, in lymph follicles, and the rest of the epithelium (Fig. 5b, c). By use of qRT-PCR of total ileal tissue, no differences in *CB*_*1*_ RNA levels were found between healthy and LPS-treated animals (Fig. 2e). Only a small amount of CD3^+^ cells co-localized with *CB*_*1*_ (Fig. 5d, arrows) which was slightly reduced after LPS treatment (Fig. 5e, f). *CB*_*2*_ mRNA staining was only marginal in the myenteric plexus (Fig. 6a) but was detectable in lymph follicles co-localizing with CD3^+^, though only with a weak ISH signal (Fig. 6b); however, *CB*_*2*_ mRNA was also seen in CD3^+^ cells of the lamina propria (Fig. 6d, e) and in F4/80^+^ cells of healthy (Fig. 6f) and LPS-treated animals (Fig. 6g). No significant differences in *CB*_*2*_ gene expression exist between healthy and LPS-treated animals as shown by qRT-PCR (Fig. 2f) and by quantification in CD3^+^ cells (Fig. 6c).

##### *MGL* gene expression is reduced in ileum of LPS-treated mice

**Fig. 7:**
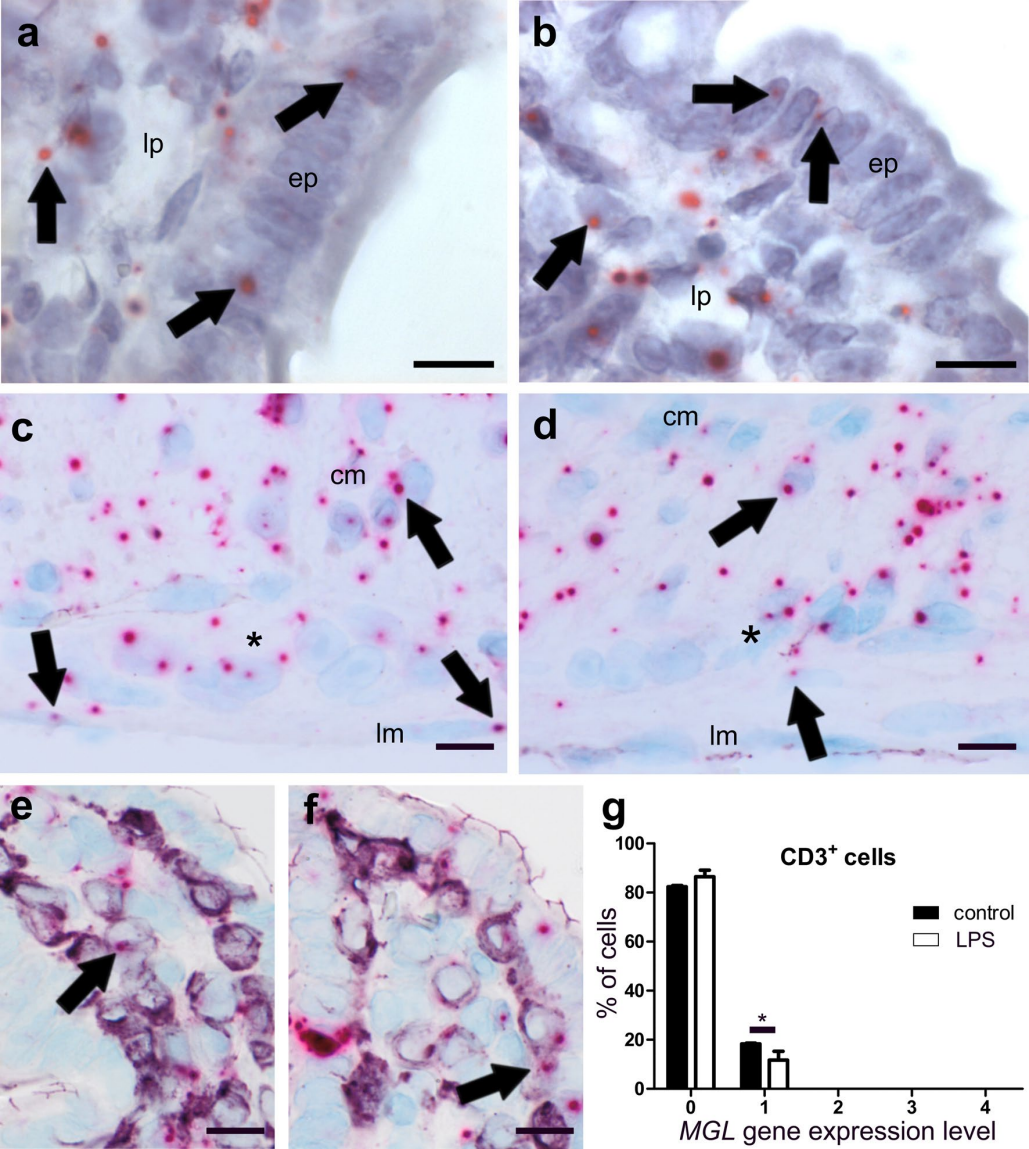
ISH staining of *MGL* mRNA (FastRed) in ileum of healthy (**a, c, e**) and LPS-treated (**b, d, f**) mice. *MGL* mRNA is equally present in epithelium (*ep*) and lamina propria (*lp*) cells of healthy ileum (**a**) and ileum from LPS-treated mice (**b** arrows denote representative mRNA staining). Expression is also present in myenteric ganglia (denoted by an asterisk) of healthy (**c**) and LPS-treated (**d**) mice. Expression of the *MGL* gene is widespread in the circular (*cm*) and the longitudinal muscle layer (*lm*), (arrows in **c**), and it is slightly decreased in LPS-treated mice (**d**). Only low co-localization of *MGL* gene expression with CD3^+^ cells (purple, VIP) is found in healthy (**e**) and even lower co-localization in LPS-treated (**f**) mice. Quantification of *MGL*/CD3 double staining is shown in **g**. Calibration bars: 10 µm; *cm* circular muscle layer, *ep* epithelium, *lp* lamina propria, *lm* longitudinal muscle layer, *cl* crypt lumen; *, myenteric ganglion

*MGL* mRNA staining was detected in the epithelium, lamina propria cells (Fig. 7a, b), myenteric ganglia and the muscle layers (Fig. 7c, d). Total gene expression of *MGL*, as determined by qRT-PCR, was decreased in the ileum of LPS-treated versus healthy animals (Fig. 2g). This corroborates the observation that staining of *MGL* mRNA in muscle layers is lower in LPS-treated (Fig. 7d) versus healthy mice (Fig. 7c). In addition, CD3^+^ cells showed a slight reduction of already low levels of *MGL* gene expression (Fig. 7e–g). For quantification in CD3^+^ cells, see Fig. 7g. No differences were found between F4/80^+^ cells of healthy (Fig. 7e) and LPS-treated mice (Fig. 7f).

### *GPR55* mRNA highly co-localizes with T-cell and macrophage markers in the ileum of healthy and LPS-treated mice

**Fig. 8:**
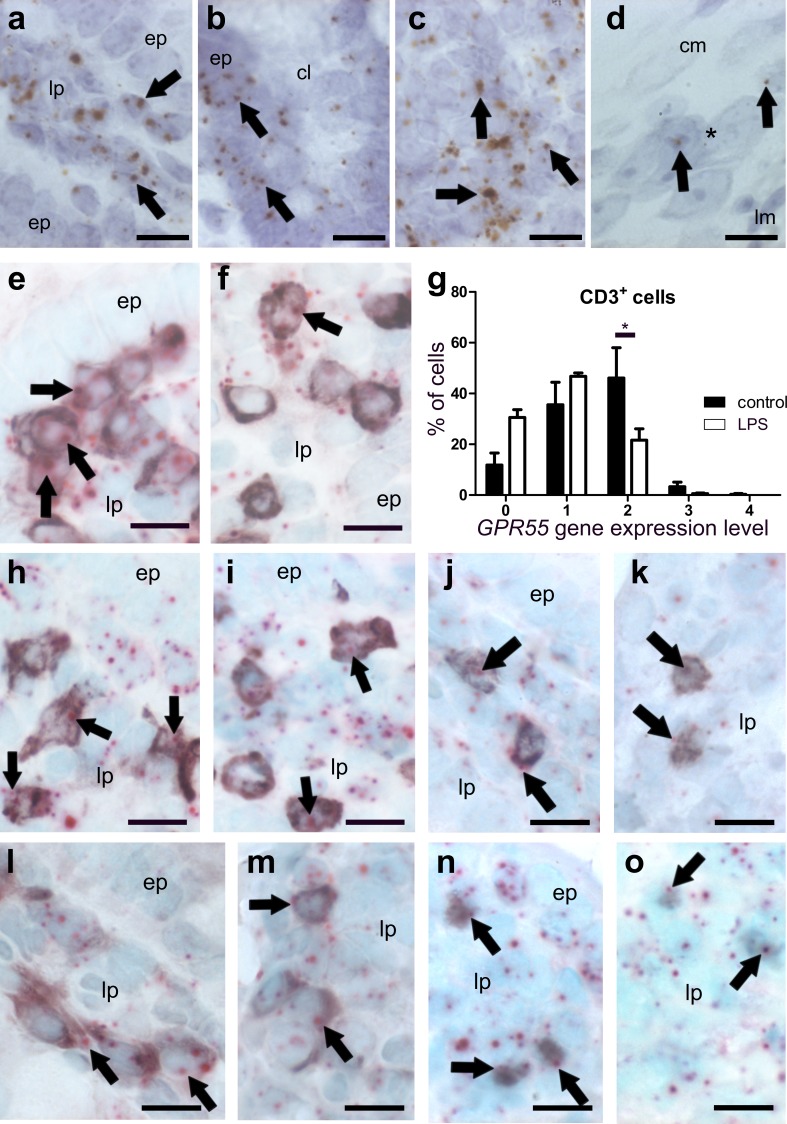
ISH staining of *GPR55* mRNA (brown, DAB, **a**–**d**) and co-localization of *GPR55* mRNA (FastRed, **e**–**o**) with markers for T-lymphocytes and macrophages (all purple, VIP) in the ileum of healthy (**a**–**d, e, h, j, l, n**) and LPS-treated mice (**f, i, k, m, o**). *GPR55* mRNA is predominantly found in the lamina propria of the ileum mucosa (*arrows* point at representative expression) and in epithelial cells of healthy mice (**a, b**). Epithelial cells (*ep*) of villi show low levels of *GPR55* mRNA (**a**), whereas crypt epithelial cells (*ep*) display relatively strong *GPR55* gene expression (**b**). In healthy animals, strong *GPR55* gene expression is detected also in lymph follicles (**c**), while in myenteric ganglia, expression is low (**d**; arrows; ganglion denoted by asterisk). *GPR55* gene expression appears to be stronger in CD3^+^ T-cells of the lamina propria (*lp*) of the healthy ileum (**e**) when compared to LPS-treated animals (**f**). Arrows denote representative staining. Quantification of *GPR55* mRNA in CD3^+^ cells is shown in **g**. In the lamina propria of the ileum mucosa, *GPR55* gene expression is seen in CD4^+^ cells (**h**, healthy; **i**, LPS-treated), CD8^+^ cells (**j**, healthy; **k**, LPS-treated), F4/80^+^ cells (**l**, healthy; **m**, LPS-treated), and FoxP3^+^ T regulatory cells (**n**, healthy; **o**, LPS-treated). Calibration bars: 10 µm; *ep* epithelium, *lp* lamina propria, *, myenteric ganglion; arrows point at representative cells expressing the *GPR55* gene

In healthy mice, strong *GPR55* mRNA staining was found in the ileal lamina propria (Fig. 8a), crypt epithelial cells (Fig. 8b) and lymph follicles (Fig. 8c). Levels of *GPR55* mRNA seemed to be lower in ileal villi (Fig. 8a) than crypts (Fig. 8b). Very low expression was detected in neurons of myenteric ganglia (Fig. 8d). *GPR55* ISH was combined with IHC to determine specific immune cells and investigate possible gene expression changes within immune cells. Like in the colon, there was a strong expression of the *GPR55* gene in CD3^+^ cells (Fig. 8e, f) which could be also detected in all investigated T-cell subpopulations (Fig. 8h–k, n, o) and F4/80^+^ cells (Fig. 8l, m).

### *GPR55* is markedly reduced in CD3^+^ cells in LPS-treated mice

Although qRT-PCR of ileal tissue from LPS-treated mice showed significant expressional changes only for the *MGL* gene but not for the other genes investigated, the detailed cellular scoring of *GPR55* revealed that in contrast to DSS-induced colitis, gene expression of *GPR55* in the ileum was lowered in CD3^+^ cells (see graph in Fig. 8), but did not change in F4/80^+^ cells (not shown).

## Discussion

Several surveys performed in recent years have indicated that people suffering from inflammatory bowel disease (IBD) are using *Cannabis* to relieve pain and other symptoms associated with the disease [rev. in (Hasenoehrl et al. 2017)]. A small clinical trial in which *Cannabis* caused moderate symptom relief in Crohn’s patients seems to confirm this (Naftali et al. 2013). Other than these reports, little evidence yet exists whether *Cannabis* or cannabinoids are protective against IBD in humans. In contrast, a high number of reports describe beneficial effects of cannabinoids in preclinical models of intestinal inflammation [rev. in (Hasenoehrl et al. 2016)].

In our study, we used ISH RNAscope^®^ combined with IHC to identify and quantify gene expression of receptors and enzymes of the ECS in cells of the ileal and colonic wall of mice exposed to DSS and LPS. It is commonly believed that the ECS is an entity that controls homeostasis in the gut and that levels of its receptors are thought to increase during an inflammatory attack. The goal was to measure gene expression of *CB*_*1*_, *CB*_*2*_, *GPR55* and *MGL* and to delineate the cell types responding to the inflammation. The ISH probes against *CB*_*1*_, *CB*_*2*_, *GPR55* and *MGL* were all tested in the respective knockout mouse providing a strong proof for the specificity of the probes, something which is missing for many antibodies used against cannabinoid receptors.

### *CB*_*1*_ and *CB*_*2*_ mRNA in the colon and the ileum

First, we showed detailed localization of these receptors in the bowel wall, confirming that CB_1_ is largely present in enteric neurons (Duncan et al. 2005) and only moderately in the epithelial lining. *CB*_*1*_ mRNA was also detectable in the longitudinal muscle layer (but hardly in the circular layer) where it does not co-localize with synaptophysin. Extra-neuronal localization of *CB*_*1*_ mRNA in the longitudinal muscle layer, therefore, is of interest with regard to a recent article that describes myogenic, besides neurogenic, relaxant effects of CB_1_ agonists in longitudinal muscle myenteric plexus preparations (LMMP) of the guinea pig (Donnerer and Liebmann 2018). qRT-PCR of whole colonic segments and quantification of *CB*_*1*_/*CB*_*2*_ mRNA in single cells of ISH/IHC stainings revealed little differences between control and DSS- or LPS-treated animals. In the latter, *CB*_*1*_ gene expression in CD3^+^ cells was even more reduced compared to an already very low expression in healthy mice (Fig. 5f). Although CB_1_ receptors are important in the regeneration of epithelial cells (Wright et al. 2005), upregulation of CB_1_ may not be necessary for the regenerative process. It is also possible that a later time point than the one chosen in our experiments might have been more accurate to measure *CB*_*1*_ mRNA, as regeneration and re-epithelization after a bout of DSS may take several weeks (Perse and Cerar 2012). A recent study also highlighted that the presence of CB_1_ in epithelial cells is crucial for the regulation of intestinal permeability (Karwad et al. 2017). However, it is not yet clear how CB_1_ contributes to permeability during inflammation because activation of CB_1_ by anandamide and 2-AG, applied to the basolateral membrane of Caco-2 cells, have shown opposing effects (Karwad et al. 2017).

The CB_2_ receptor showed only little gene expression in myenteric neurons and the epithelium of the colon. However, mouse models of colitis show improvement in the presence of CB_2_ through apoptosis of T-cells (Singh et al. 2012) and through inhibition of the NLRP3 inflammasome in macrophages (Ke et al. 2016). A role in the protection of CB_2_ expressing immune cells against inflammation is, therefore, quite likely. As observed in the qRT-PCR, the presence of *CB*_*2*_ mRNA was not increased in whole bowel wall of the DSS and LPS model in comparison with the untreated animals. In contrast, *CB*_*2*_ mRNA was described to be upregulated in a TNBS colitis model (Storr et al. 2009). This discrepancy may lie in the different models that were used. Unlike the DSS model, the TNBS colitis model is caused by an immune response to the hapten TNBS, and therefore, differs from the DSS model, which is bacterially driven (Eichele and Kharbanda 2017). It is unclear if and how the strong presence of CB_2_ in B-cells found in our study could contribute to an amelioration of the inflammation because the presence of T- and B-cells were previously thought not to be needed for the development of acute DSS colitis (Dieleman et al. 1994). However, later studies showed that the CB_2_ receptor is critical for the formation of T- and B-cells (Ziring et al. 2006), and also for the homing and retention of marginal zone B lineage cells (Basu et al. 2011). More recent reports have revealed that B cells can protect from DSS colitis in cooperation with Tregs (Wang et al. 2015). CB_2_ receptors on B cells, therefore, could participate in the protection against intestinal inflammation.

### *GPR55* mRNA in the colon and the ileum

By use of a GPR55 antagonist and GPR55^−/−^ mice, we previously detected a pro-inflammatory role of GPR55 in DSS and TNBS inflammation models (Stancic et al. 2015). In the present study, we also focused on GPR55 in immune cells. We observed that mRNA levels of *GPR55* in whole colon segments were diminished in DSS mice as compared to controls while in LPS-treated mice, no significant changes were noticed. In line with previous results (Li et al. 2013), we also observed that *GPR55* mRNA levels were higher in the ileum than in the colon (online resource Fig. 1d). Unlike *CB*_*1*_, *GPR55* mRNA showed the prominent presence in epithelial cells, and even more in lamina propria cells, such as macrophages and T-cells. But like *CB*_*2*_ (and contrary to *CB*_*1*_), the *GPR55* gene is only little expressed in the myenteric plexus. Most changes, i.e., reductions, in *GPR55* gene expression were observed in T-cells during LPS-induced inflammation, while in DSS-induced inflammation, a reduction in *GPR55* gene expression was predominant rather in F4/80^+^ cells. This is well in line with previous results we obtained in the azoxymethane + DSS cancer model, in which genetic knockout of GPR55 led to a substantially altered T- and myeloid-derived suppressor cell (MDSC) profile when compared to wild-type mice (Hasenoehrl et al. 2018). Additionally, we could previously show that treatment with a GPR55 antagonist leads to reduced influx of lymphocytes and macrophages into the inflamed colon in the DSS model (Stancic et al. 2015). The GPR55 receptor, therefore, seems to primarily act in immune cells during inflammatory attacks in the GI tract.

### Presence of *MGL* mRNA

MGL is a key enzyme in the degradation of 2-AG, and therefore, a well characterized member of the ECS (Grabner et al. 2017). Pharmacologic inhibition of MGL has been shown to improve TNBS-induced colitis (Alhouayek et al. 2011). Inhibition of MGL also protects from LPS-induced lung injury (Costola-de-Souza et al. 2013). We found that the presence of *MGL* mRNA was decreased in DSS mice and after LPS treatment, and since MGL inhibition protects from injury, a decrease in MGL in the disease models could very well be part of a protective mechanism. Contrary to previous reports (Duncan et al. 2008), we observed *MGL* gene expression in the smooth muscle layer of the GI tract, indicating that *MGL* is expressed in smooth muscle cells themselves although the presence in resident macrophages cannot be excluded (Wehner and Engel 2017). However, owing to the abundance of *MGL* gene expression seen in the longitudinal muscle layer which is devoid of resident macrophages (Wehner and Engel 2017) as well as the observation that most of *MGL* mRNA does not co-localize with synaptophysin (Fig. 3e), *MGL* is likely expressed in the smooth myocytes themselves.

To conclude, RNAscope^®^ ISH technique combined with IHC is an extremely valuable method for identifying gene expression and regulation of receptors and enzymes at a single cell basis in situ, as demonstrated by the response of the ECS to intestinal and systemic inflammation. Alterations in the gene expression of *GPR55* and *MGL* in CD3^+^ lymphocytes and F4/80^+^ macrophages indicate that these two components of the ECS are an important part of the innate and adaptive immune response to the inflammatory attack. An upregulation of *CB*_*1*_ and *CB*_*2*_, often suggested as part of a homeostatic response of the ECS to injury, was not observed.

## Electronic supplementary material

Below is the link to the electronic supplementary material.

Supplementary material 1 (PDF 1143 KB)

## References

[CR1] Acharya N, Penukonda S, Shcheglova T, Hagymasi AT, Basu S, Srivastava PK (2017). Endocannabinoid system acts as a regulator of immune homeostasis in the gut. Proc Natl Acad Sci USA.

[CR2] Alhouayek M, Lambert DM, Delzenne NM, Cani PD, Muccioli GG (2011). Increasing endogenous 2-arachidonoylglycerol levels counteracts colitis and related systemic inflammation. FASEB J.

[CR3] Baek JH, Darlington CL, Smith PF, Ashton JC (2013). Antibody testing for brain immunohistochemistry: brain immunolabeling for the cannabinoid CB(2) receptor. J Neurosci Methods.

[CR4] Basu S, Ray A, Dittel BN (2011). Cannabinoid receptor 2 is critical for the homing and retention of marginal zone B lineage cells and for efficient T-independent immune responses. J Immunol.

[CR5] Cani PD, Plovier H, Van Hul M, Geurts L, Delzenne NM, Druart C, Everard A (2016). Endocannabinoids—at the crossroads between the gut microbiota and host metabolism. Nat Rev Endocrinol.

[CR6] Cecyre B, Thomas S, Ptito M, Casanova C, Bouchard JF (2014). Evaluation of the specificity of antibodies raised against cannabinoid receptor type 2 in the mouse retina. Naunyn Schmiedebergs Arch Pharmacol.

[CR7] Costola-de-Souza C, Ribeiro A, Ferraz-de-Paula V, Calefi AS, Aloia TP, Gimenes-Junior JA, de Almeida VI, Pinheiro ML, Palermo-Neto J (2013). Monoacylglycerol lipase (MAGL) inhibition attenuates acute lung injury in mice. PLoS One.

[CR8] Di Marzo V, Piscitelli F (2015). The endocannabinoid system and its modulation by phytocannabinoids. Neurotherapeutics.

[CR9] Dieleman LA, Ridwan BU, Tennyson GS, Beagley KW, Bucy RP, Elson CO (1994). Dextran sulfate sodium-induced colitis occurs in severe combined immunodeficient mice. Gastroenterology.

[CR10] Donnerer J, Liebmann I (2018). Effect of CB1 Ligands on Neurogenic and Myogenic Contractile Responses in the Guinea-Pig Ileum. Pharmacology.

[CR11] Duncan M, Davison JS, Sharkey KA (2005). Review article: endocannabinoids and their receptors in the enteric nervous system. Aliment Pharmacol Ther.

[CR12] Duncan M, Thomas AD, Cluny NL, Patel A, Patel KD, Lutz B, Piomelli D, Alexander SP, Sharkey KA (2008). Distribution and function of monoacylglycerol lipase in the gastrointestinal tract. Am J Physiol Gastrointest Liver Physiol.

[CR13] Eichele DD, Kharbanda KK (2017). Dextran sodium sulfate colitis murine model: An indispensable tool for advancing our understanding of inflammatory bowel diseases pathogenesis. World J Gastroenterol.

[CR14] Farzi A, Reichmann F, Meinitzer A, Mayerhofer R, Jain P, Hassan AM, Frohlich EE, Wagner K, Painsipp E, Rinner B, Holzer P (2015). Synergistic effects of NOD1 or NOD2 and TLR4 activation on mouse sickness behavior in relation to immune and brain activity markers. Brain Behav Immun.

[CR15] Galiazzo G, Giancola F, Stanzani A, Fracassi F, Bernardini C, Forni M, Pietra M, Chiocchetti R (2018). Localization of cannabinoid receptors CB1, CB2, GPR55, and PPARalpha in the canine gastrointestinal tract. Histochem Cell Biol.

[CR16] Grabner GF, Zimmermann R, Schicho R, Taschler U (2017). Monoglyceride lipase as a drug target: At the crossroads of arachidonic acid metabolism and endocannabinoid signaling. Pharmacol Ther.

[CR17] Grimsey NL, Goodfellow CE, Scotter EL, Dowie MJ, Glass M, Graham ES (2008). Specific detection of CB1 receptors; cannabinoid CB1 receptor antibodies are not all created equal!. J Neurosci Methods.

[CR18] Han X, Fink MP, Yang R, Delude RL (2004). Increased iNOS activity is essential for intestinal epithelial tight junction dysfunction in endotoxemic mice. Shock.

[CR19] Hasenoehrl C, Taschler U, Storr M, Schicho R (2016). The gastrointestinal tract - a central organ of cannabinoid signaling in health and disease. Neurogastroenterol Motil.

[CR20] Hasenoehrl C, Storr M, Schicho R (2017). Cannabinoids for treating inflammatory bowel diseases: where are we and where do we go?. Expert Rev Gastroenterol Hepatol.

[CR21] Hasenoehrl C, Feuersinger D, Sturm EM, Barnthaler T, Heitzer E, Graf R, Grill M, Pichler M, Beck S, Butcher L, Thomas D, Ferreiros N, Schuligoi R, Schweiger C, Haybaeck J, Schicho R (2018). G protein-coupled receptor GPR55 promotes colorectal cancer and has opposing effects to cannabinoid receptor 1. Int J Cancer.

[CR22] Karwad MA, Couch DG, Theophilidou E, Sarmad S, Barrett DA, Larvin M, Wright KL, Lund JN, O’Sullivan SE (2017). The role of CB1 in intestinal permeability and inflammation. FASEB J.

[CR23] Ke P, Shao BZ, Xu ZQ, Wei W, Han BZ, Chen XW, Su DF, Liu C (2016). Activation of cannabinoid receptor 2 ameliorates DSS-induced colitis through inhibiting NLRP3 inflammasome in macrophages. PLoS One.

[CR24] Kimura H, Sawada N, Tobioka H, Isomura H, Kokai Y, Hirata K, Mori M (1997). Bacterial lipopolysaccharide reduced intestinal barrier function and altered localization of 7H6 antigen in IEC-6 rat intestinal crypt cells. J Cell Physiol.

[CR25] Li Y, Kim J (2015). Neuronal expression of CB2 cannabinoid receptor mRNAs in the mouse hippocampus. Neuroscience.

[CR26] Li K, Fichna J, Schicho R, Saur D, Bashashati M, Mackie K, Li Y, Zimmer A, Goke B, Sharkey KA, Storr M (2013). A role for O-1602 and G protein-coupled receptor GPR55 in the control of colonic motility in mice. Neuropharmacology.

[CR27] Lin XH, Yuece B, Li YY, Feng YJ, Feng JY, Yu LY, Li K, Li YN, Storr M (2011). A novel CB receptor GPR55 and its ligands are involved in regulation of gut movement in rodents. Neurogastroenterol Motil.

[CR28] Liu C, Li A, Weng YB, Duan ML, Wang BE, Zhang SW (2009). Changes in intestinal mucosal immune barrier in rats with endotoxemia. World J Gastroenterol.

[CR29] Marchalant Y, Brownjohn PW, Bonnet A, Kleffmann T, Ashton JC (2014). Validating Antibodies to the Cannabinoid CB2 Receptor: Antibody Sensitivity Is Not Evidence of Antibody Specificity. J Histochem Cytochem.

[CR30] Naftali T, Bar-Lev Schleider L, Dotan I, Lansky EP, Sklerovsky Benjaminov F, Konikoff FM (2013). Cannabis induces a clinical response in patients with Crohn’s disease: a prospective placebo-controlled study. Clin Gastroenterol Hepatol.

[CR31] Perse M, Cerar A (2012) Dextran sodium sulphate colitis mouse model: traps and tricks. J Biomed Biotechnol 2012:718617 10.1155/2012/718617 PMC3361365 22665990

[CR32] Pertwee RG (2015). Endocannabinoids and Their Pharmacological Actions. Handb Exp Pharmacol.

[CR33] Schicho R, Storr M (2010). Targeting the endocannabinoid system for gastrointestinal diseases: future therapeutic strategies. Expert Rev Clin Pharmacol.

[CR34] Shi X (2010). Resident macrophages in the cochlear blood-labyrinth barrier and their renewal via migration of bone-marrow-derived cells. Cell Tissue Res.

[CR35] Singh UP, Singh NP, Singh B, Price RL, Nagarkatti M, Nagarkatti PS (2012). Cannabinoid receptor-2 (CB2) agonist ameliorates colitis in IL-10(–/–) mice by attenuating the activation of T cells and promoting their apoptosis. Toxicol Appl Pharmacol.

[CR36] Stancic A, Jandl K, Hasenohrl C, Reichmann F, Marsche G, Schuligoi R, Heinemann A, Storr M, Schicho R (2015). The GPR55 antagonist CID16020046 protects against intestinal inflammation. Neurogastroenterol Motil.

[CR37] Storr MA, Keenan CM, Zhang H, Patel KD, Makriyannis A, Sharkey KA (2009). Activation of the cannabinoid 2 receptor (CB2) protects against experimental colitis. Inflamm Bowel Dis.

[CR38] Sumida H, Lu E, Chen H, Yang Q, Mackie K, Cyster JG (2017) GPR55 regulates intraepithelial lymphocyte migration dynamics and susceptibility to intestinal damage. Sci Immunol 2:10.1126/sciimmunol.aao1135 10.1126/sciimmunol.aao1135 PMC5847323 29222090

[CR39] Taschler U, Radner FP, Heier C, Schreiber R, Schweiger M, Schoiswohl G, Preiss-Landl K, Jaeger D, ReiterKoefeler BHC, Wojciechowski J, Theussl C, Penninger JM, Lass A, Haemmerle G, Zechner R, Zimmermann R (2011). Monoglyceride lipase deficiency in mice impairs lipolysis and attenuates diet-induced insulin resistance. J Biol Chem.

[CR40] Wang F, Flanagan J, Su N, Wang LC, Bui S, Nielson A, Wu X, Vo HT, Ma XJ, Luo Y (2012). RNAscope: a novel in situ RNA analysis platform for formalin-fixed, paraffin-embedded tissues. J Mol Diagn.

[CR41] Wang L, Ray A, Jiang X, Wang JY, Basu S, Liu X, Qian T, He R, Dittel BN, Chu Y (2015). T regulatory cells and B cells cooperate to form a regulatory loop that maintains gut homeostasis and suppresses dextran sulfate sodium-induced colitis. Mucosal Immunol.

[CR42] Wehner S, Engel DR (2017). Resident macrophages in the healthy and inflamed intestinal muscularis externa. Pflugers Arch.

[CR43] Wright K, Rooney N, Feeney M, Tate J, Robertson D, Welham M, Ward S (2005). Differential expression of cannabinoid receptors in the human colon: cannabinoids promote epithelial wound healing. Gastroenterology.

[CR44] Wright KL, Duncan M, Sharkey KA (2008). Cannabinoid CB2 receptors in the gastrointestinal tract: a regulatory system in states of inflammation. Br J Pharmacol.

[CR45] Ziring D, Wei B, Velazquez P, Schrage M, Buckley NE, Braun J (2006). Formation of B and T cell subsets require the cannabinoid receptor CB2. Immunogenetics.

