# Supplementary material for: Cellular localization and regulation of receptors and enzymes of the endocannabinoid system in intestinal and systemic inflammation
Source: Histochem Cell Biol. 2018 Sep 8;151(1):5–20. doi: 10.1007/s00418-018-1719-0 (PMC6328631; doi:10.1007/s00418-018-1719-0)

Online resources, supplementary material to the article:

Cellular localization and regulation of receptors and enzymes of the endocannabinoid system in intestinal and systemic inflammation

Journal: Histochemistry and Cell Biology

Authors: [1] Magdalena Grill, [1] Carina Hasenoehrl, [1,2] Melanie Kienzl, [1] Julia Kargl, [1,2,*] Rudolf Schicho

Affiliation: [1] Otto Loewi Research Center, Pharmacology Section, Medical University of Graz, Graz, Austria; [2] BioTechMed Graz, Austria

*Corresponding author: Rudolf Schicho (rudolf.schicho@medunigraz.at)

Online resource **Fig. 1**:

Representative immunohistochemical staining of GPR55 using polyclonal rabbit anti-GPR55 antibody (1:500, Cayman Chemicals, Ann Arbor, MI, USA) in murine wildtype (**a**) and GPR55$^{-/-}$ (**b**) colon tissue; note unspecific staining in the GPR55$^{-/-}$ tissue. (**c**) Testing of several anti-GPR55 antibodies in colon tissue of wildtype (WT1-WT7) and GPR55 $^{-/-}$ (knockout, KO1-KO7) mice by SDS-Page/ Western blot analysis (used antibodies: Thermo Sc: rabbit anti-GPR55 antibody, ThermoFisher Scientific, Waltham, MA, USA; Abcam: polyclonal rabbit anti-GPR55 antibody, Abcam, Cambridge, UK; Cayman: polyclonal rabbit anti-GPR55 Ab, Cayman Chemicals; Genetex 1 and 2: polyclonal rabbit anti-GPR55 antibody, GeneTex International Corporation, Irvine, CA, USA). (**d**) Comparison of CB_1, CB_2, *GPR55* and *MGL* gene expression in murine colon and ileum by qRT-PCR.

Online resource **Fig. 2**:

ISH RNAscope® staining of CB_1 (**a, b**), CB_2 (**c, d**), *MGL* (**e, f**) and *GPR55* (**g, h**) in wildtype (**a, c, e, g**) and corresponding knockout mice (**b, d, f, h**). Inserts show higher magnification of relevant region with high expression of the target gene in wildtype tissue. Calibration bar low magnification: 20 µm, Insert: 10 µm.

Online resource Fig. 1

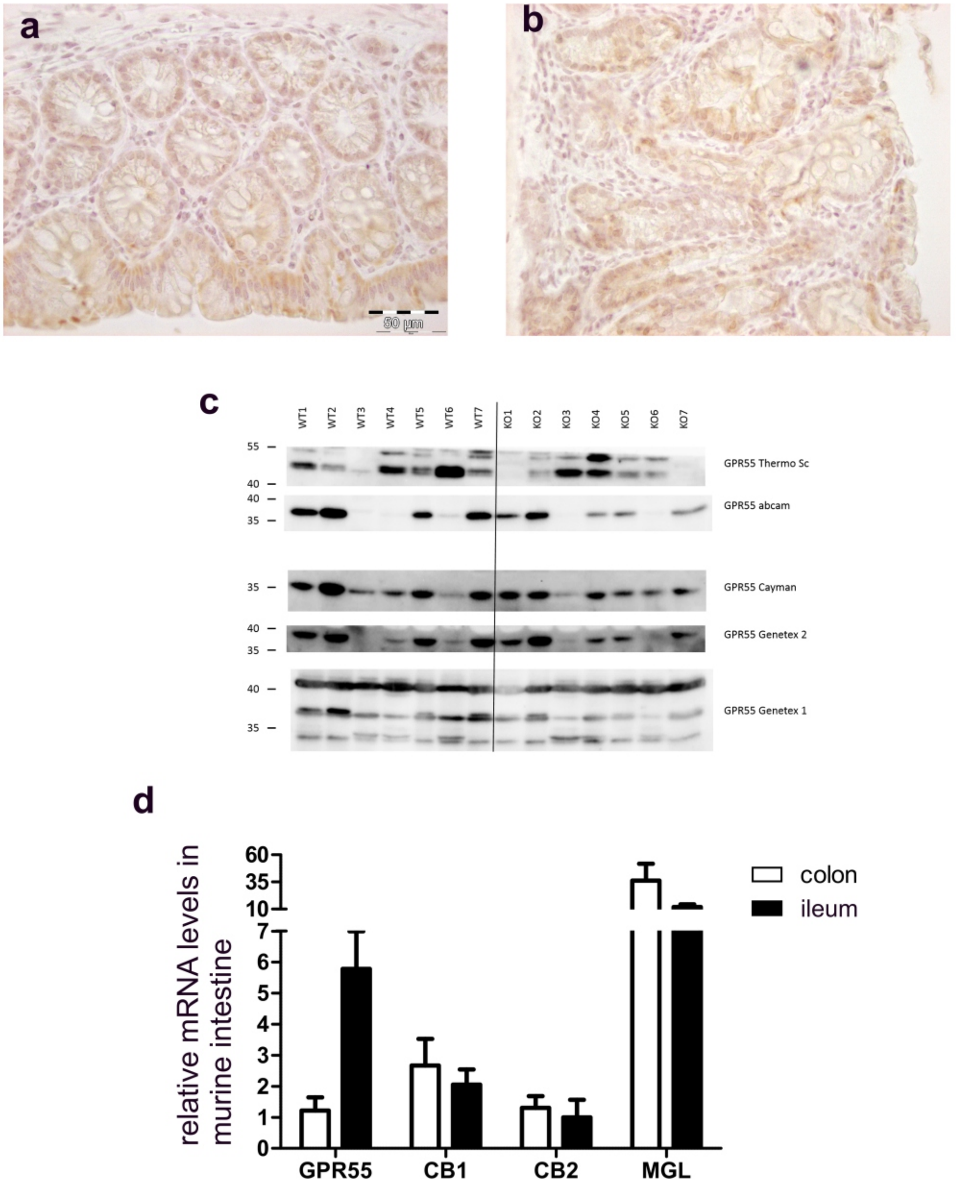

Online resource Fig. 2

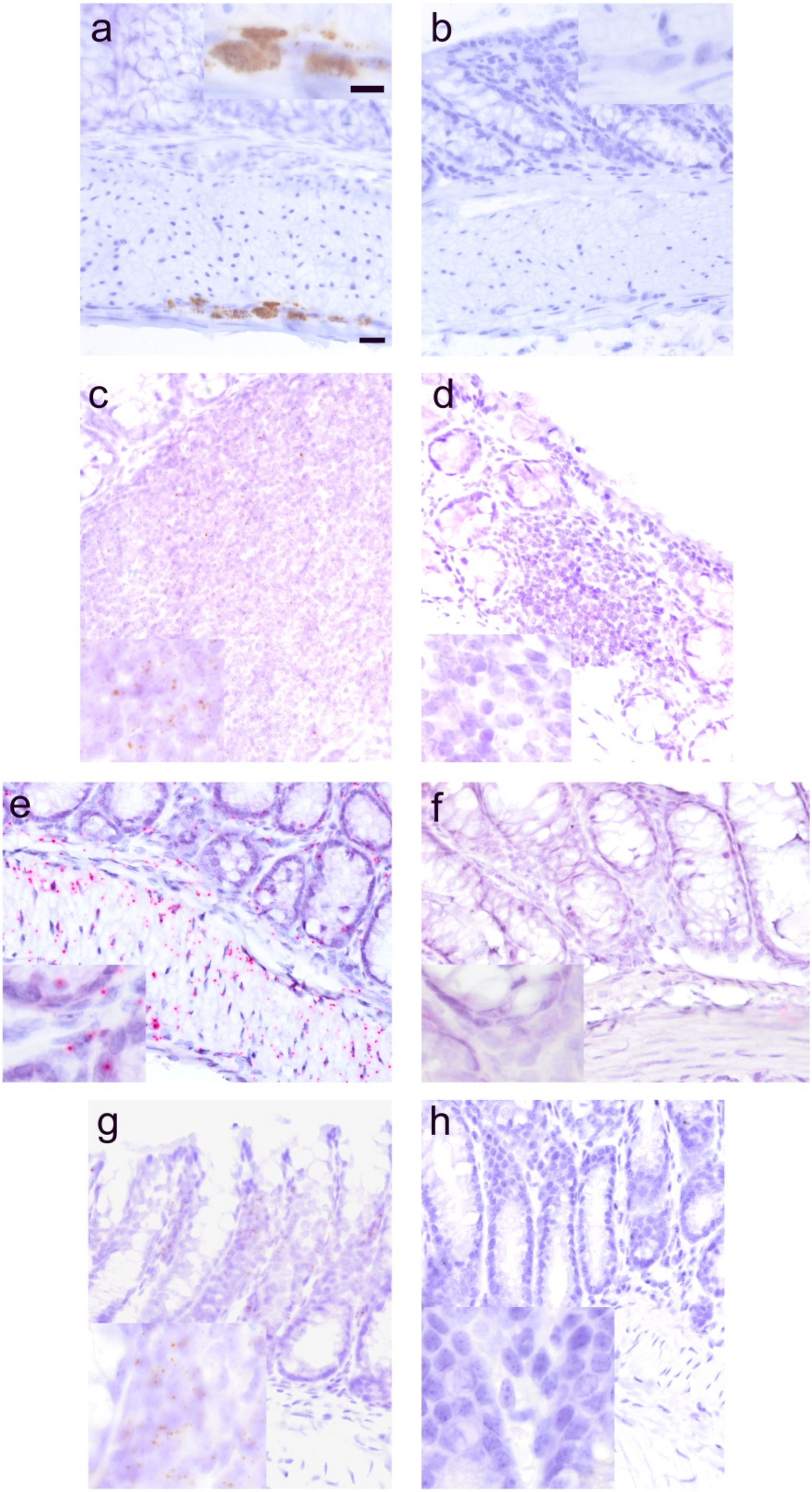

Supplement: Supplementary file 1 — Supplementary material 1 (PDF 1143 KB) [file 418_2018_1719_MOESM1_ESM.pdf]
